# Plasma Concentrations of Endocannabinoids and Related Primary Fatty Acid Amides in Patients with Post-Traumatic Stress Disorder

**DOI:** 10.1371/journal.pone.0062741

**Published:** 2013-05-07

**Authors:** Daniela Hauer, Gustav Schelling, Hannah Gola, Patrizia Campolongo, Julia Morath, Benno Roozendaal, Gilava Hamuni, Alexander Karabatsiakis, Piray Atsak, Michael Vogeser, Iris-Tatjana Kolassa

**Affiliations:** 1 Department of Anaesthesiology, Ludwig-Maximilians-University, Munich, Germany; 2 Department of Clinical and Biological Psychology, Institute of Psychology and Education, University of Ulm, Ulm, Germany; 3 Department of Physiology and Pharmacology, Sapienza University of Rome, Rome, Italy; 4 Department of Clinical and Neuropsychology, Department of Psychology, University of Konstanz, Konstanz, Germany; 5 Department of Cognitive Neuroscience, Radboud University Nijmegen Medical Centre and Donders Institute for Brain, Cognition and Behaviour, Radboud University, Nijmegen, The Netherlands; 6 Department of Clinical Chemistry, Ludwig-Maximilians-University, Munich, Germany; University of Wuerzburg, Germany

## Abstract

**Background:**

Endocannabinoids (ECs) and related N-acyl-ethanolamides (NAEs) play important roles in stress response regulation, anxiety and traumatic memories. In view of the evidence that circulating EC levels are elevated under acute mild stressful conditions in humans, we hypothesized that individuals with traumatic stress exposure and post-traumatic stress disorder (PTSD), an anxiety disorder characterized by the inappropriate persistence and uncontrolled retrieval of traumatic memories, show measurable alterations in plasma EC and NAE concentrations.

**Methods:**

We determined plasma concentrations of the ECs anandamide (ANA) and 2-arachidonoylglycerol (2-AG) and the NAEs palmitoylethanolamide (PEA), oleoylethanolamide (OEA), stearoylethanolamine (SEA), and N-oleoyldopamine (OLDA) by HPLC-MS-MS in patients with PTSD (n = 10), trauma-exposed individuals without evidence of PTSD (n = 9) and in healthy control subjects (n = 29). PTSD was diagnosed according to DSM-IV criteria by administering the *Clinician Administered PTSD Scale* (CAPS), which also assesses traumatic events.

**Results:**

Individuals with PTSD showed significantly higher plasma concentrations of ANA (0.48±0.11 vs. 0.36±0.14 ng/ml, p = 0.01), 2-AG (8.93±3.20 vs. 6.26±2.10 ng/ml, p<0.01), OEA (5.90±2.10 vs. 3.88±1.85 ng/ml, p<0.01), SEA (2.70±3.37 vs. 0.83±0.47, ng/ml, p<0.05) and significantly lower plasma levels of OLDA (0.12±0.05 vs. 0.45±0.59 ng/ml, p<0.05) than healthy controls. Moreover, PTSD patients had higher 2-AG plasma levels (8.93±3.20 vs. 6.01±1.32 ng/ml, p = 0.03) and also higher plasma concentrations of PEA (4.06±1.87 vs. 2.63±1.34 ng/ml, p<0.05) than trauma-exposed individuals without evidence of PTSD. CAPS scores in trauma-exposed individuals with and without PTSD (n = 19) correlated positively with PEA (*r* = 0.55, p = 0.02) and negatively with OLDA plasma levels (*r* = −0.68, p<0.01). CAPS subscores for intrusions (*r* = −0.65, p<0.01), avoidance (*r* = −0.60, p<0.01) and hyperarousal (*r* = −0.66, p<0.01) were all negatively related to OLDA plasma concentrations.

**Conclusions:**

PTSD appears to be associated with changes in plasma EC/NAE concentrations. This may have pathophysiological and diagnostic consequences but will need to be reproduced in larger cohorts.

## Introduction

Endocannabinoids (ECs) and related N-acyl-ethanolamides (NAEs) play an essential role in many physiological processes, including energy metabolism, immune function and both central and peripheral nervous system function [1]. They are also implicated in a considerable number of pathophysiological conditions such as cardiovascular disorders [2], systemic inflammation and neuroinflammation [3], [4], obesity and the metabolic syndrome [5], cancer [6] and mental disorders such as depression [7] and schizophrenia [8].The best investigated ECs are anandamide (ANA) and 2-arachidonoylglycerol (2-AG) which both bind to CB1 and CB2 receptors. However, there are a number of other biologically highly active molecules which also have been linked to the EC system and show stress or memory-modulating capabilities. These compounds include palmitoylethanolamide (PEA), oleoylethanolamide (OEA), stearoylethanolamide (SEA), N-oleoyldopamine (OLDA) and a number of other NAEs [8].

Despite the abovementioned diversity in central and peripheral functions, recent studies in animals [9] and humans [10] point to a major role of EC signaling in both the periphery [10] and central nervous system [11] in controlling adaptive processes to aversive situations and regulating and limiting stress reactions. As part of this protective role under aversive conditions, elevated EC signaling during stress has been shown to dampen the development of anxiety [12] and to regulate the encoding [13], retrieval [14] and extinction [15] of traumatic memories. Given the fact that post-traumatic stress disorder (PTSD) is an anxiety disorder characterized by an inappropriate persistence and uncontrolled retrieval of traumatic memories, it can be assumed that changes in EC signaling might play a role in either the development or persistence of PTSD. This line of reasoning is corroborated by the observation that patients with PTSD exhibit high rates of cannabis abuse which may represent a form of self-medication [16], and a recent study demonstrated that a stimulation of EC signaling in patients with PTSD by administration of the synthetic cannabinoid nabilone resulted in an improvement of memory-related PTSD symptoms [17].

Although primary fatty acid amides are mainly active in the brain, there are a number of recent studies which demonstrated that EC and NAE blood concentrations increase in response to acute stress [7], [10] and that they are higher in individuals who show stress tolerance [10] and lower in patients with depression [18]. EC plasma concentrations are also modified by the presence of affective disorders and severe mental illness [7], [8]. Furthermore, a pilot study by our group in critically ill patients (n = 90) awaiting cardiac surgery indicated increased plasma concentrations of ANA and 2-AG in stressed patients with traumatic experiences and PTSD symptoms as a result of heart disease [19]. The presence of heart disease, however, could have influenced plasma EC concentrations [20], and this did not allow the demonstration of a direct relationship between PTSD symptoms and plasma EC levels. The current study was therefore performed in a cohort of physically more healthy subjects who consisted of 1) individuals with PTSD due to war and torture experiences, 2) trauma-exposed subjects who did not develop PTSD and 3) healthy controls. The primary hypothesis we wanted to test was that chronically stressed individuals with PTSD have increased plasma concentrations of ECs and NAEs when compared to subjects after trauma exposure alone who did not develop PTSD and also in comparison to healthy controls. Our secondary hypothesis was that EC/NAE plasma levels were positively related to the number of traumatic events reported by the subjects.

## Participants and Procedures

### Ethics Statement

All procedures were approved by the Ethical Committees of the Universities of Konstanz and Munich (protocol# 046-13 and 439-09) and were carried out in accordance with the Declaration of Helsinki 2008. Written informed consent was obtained from all individuals enrolled in the study.

### Participants

All trauma-exposed participants were diagnosed at the Center of Excellence for Psychotraumatology, University of Konstanz, Germany. We assessed 19 individuals (age: 33.2±9.8 years, 9 females) with prior trauma exposure (ten of them fulfilled DSM-IV-TR criteria for current PTSD, whereas 9 had not developed PTSD) (Table 1). Trauma-exposed participants were refugees (n = 6 from the Middle East and Afghanistan, n = 6 from Africa, n = 6 from the Balkan and n = 1 from Eastern Europe), most of them (84.2%) with a history of persecution, war and torture experiences. Trauma-exposed participants had multiple traumatic experiences with 8.8±6.9 different traumatic event types on the event checklist of the CAPS [21] as well as 8.1±2.2 war- and torture-related events. Average CAPS scores were 94.9±15.4 for the trauma-exposed subjects with PTSD and 24.6±24.4 for trauma-exposed subjects without PTSD (p<0.01, Table 1). PTSD symptoms in patients with PTSD were present between 6 and 204 months and all patients except one had experienced the traumatic events during early or late adulthood.

**Table 1:** Demographic and clinical data of the study groups.

Variable	Trauma-exposed, no PTSD (n = 9)^d^	Trauma-exposed, PTSD (n = 10)^e^	Controls (n = 29)^f^	*p*
Age^a^ (y)	33.6±12.2	33.8±7.5	33.5±9.3	0.96
Sex (f/m)	3/6	2/8	17/12	0.08
Education^a^ (y)	10.1±4.3	8.7±3.0*	13.3±3.1	0.01
Smokers (yes/no)	6/3	6/4	4/25	0.17
* Number of cigarettes/day*	2.4±4.0	7.3±11.4	1.7±5.2	0.09
Medication use (yes/no)				
* Sedatives/anxiolytics*	*0/9*	*0/10*	*0/29*	*–*
* Antidepressives* b	*2/7*	*3/7* *	*0/29*	*0.01*
* Mood stabilizers*	*1/8*	*1/9*	*0/29*	*0.14*
* Antacids*	*2/7*	*2/8*	*0/29*	*0.20*
* Anticontraceptives*	*2/7*	*0/10*	*4/25*	*0.40*
Number of traumatic event types^a^				
* War or torture events*	*5.2±5.7*	*12.0±6.5* *	*0.44±0.7* c	*<0.01*
* CAPS events*	*7.7±2.3*	*8.5±2.1* #	*2.7±1.2* c	*<0.01*
CAPS Score^a^	24.6±24.4	94.9±15.4*	0.0 c	<0.01
* CAPS – intrusions*	*10.6±9.8*	*32.8±5.5* *	*0.0*	*<0.01*
* CAPS – avoidance*	*8.4±6.5*	*36.2±8.2* *	*0.0*	*<0.01*
* CAPS - hyperarousal*	*8.4.±10.0*	*25.9±7.6* *	*0.0*	*<0.01*
HAMD Score^a^	9.9±13.5	28.4±7.8*	2.80±6.7^c^	<0.01
SOMS Score^a^	10.2±10.0	29.0±13.4*	1.8±4.0^c^	<0.01

a Data are mean±SD; (y) =  years; (f) =  female; (m) =  male.

b Trauma-exposed patients received amitriptylin (n = 2), PTSD patients mirtazapin (n = 2) and amitriptylin+mirtazapin (n = 1).

c These scores were only available for ethnically matched controls recruited by the Trauma Center of University of Konstanz (n = 9).

d Trauma-exposed individuals without PTSD were of Caucasian origin (n = 6) (two from Iran and two from Turkey, one from Bosnia and one from Afghanistan) and 3 were Black-Africans (one from Gambia, one from Eritrea and one from Senegal).

e Trauma- exposed individuals with PTSD were Caucasians (n = 8) (two from Turkey, two from Iran, one from Afghanistan, one from Syria, one from Kosovo and one from Bosnia) and two were Black-Africans (one from Nigeria and one from Togo).

f Controls were Caucasians (twenty were Germans, one each were from Turkey, Armenia, Israel, two from Romania and two were Russians) and 2 were from Africa (Eritrea and Sudan).

* Significantly higher values compared to trauma-exposed individuals *without* PTSD and to healthy controls.

# Significantly higher values compared to healthy controls.

Eight of the trauma-exposed individuals met the DSM-IV criteria for a current major depressive episode (two subjects from the trauma exposure only group and six individuals with PTSD, p = 0.15, Fisher’s exact test) and two subjects from the PTSD group fulfilled DSM-IV criteria for alcohol dependency. Seven of the 19 trauma-exposed individuals reported current intake of anxiolytic, antidepressant, neuroleptic or contraceptive medication. With the exception of antidepressives, medication use was equally distributed between traumatized individuals and those with PTSD (Table 1). Twelve subjects were cigarette smokers. Four of the trauma-exposed individuals reported chronic somatic disorders (two had recent treatment for stomach ulcerations, one had a rheumatic disorder, and one was diagnosed as hepatitis B-positive). Three of these individuals did not have PTSD and 1 belonged to the PTSD group. None of the study participants took steroids (neither orally nor by inhalation), had acute illness or organic disorders such as diabetes, atherosclerotic disease or obesity (these conditions are known to influence the EC system [20], [22]), had a history of cannabis or other drug abuse or were abusing drugs at the time of evaluation or had evidence of psychosis.

Controls were recruited by the Department of Anesthesiology of the University of Munich (n = 20) and by the Center of Psychotraumatology of the University of Konstanz (n = 9). Controls from Munich were of Western European descent and controls from Konstanz came from the Middle East (n = 6) from Russia (n = 1), Rumania (n = 1) and Africa (n = 1). Four individuals from the control group were current cigarette smokers (Table 1).

### Procedures

Trauma-exposed individuals as well as those controls who were recruited by the Centre of Excellence for Psychotraumatology underwent an extensive standardized clinical interview administered by experienced clinical psychologists and trained translators, starting always at 10∶00 h. Upon arrival at the outpatient clinic, procedures were explained and written informed consent was obtained. Subsequently, 10 ml of venous blood for EC and cortisol measurements was drawn around 10∶30 h by medical personnel with the subjects in a comfortable seating position. Afterwards, sociodemographic as well as medical information was acquired. Somatic symptoms were assessed using a shortened version of *Screening for Somatoform Symptoms-7* (SOMS-7) [23]. During the second part of the interview, participants were interviewed in a standardized manner about the presence of individual traumatic experiences using the event checklist of the CAPS and the *Vivo Checklist of War, Detention and Torture Events* [24] which assesses common traumatic experiences in conflict regions and during torture. Subsequently, PTSD symptom frequency and severity were assessed with the CAPS [21]. The *Mini International Neuropsychiatric Interview* (MINI) [25] was applied to screen for potential comorbid mental disorders. In addition, the severity of depressive symptoms was assessed with the *Hamilton Depression Rating Scale* (HAMD) [26].

Control subjects from Munich (n = 20) were interviewed on the presence or history of acute or chronic physical and mental disorders, but the abovementioned instruments for the diagnosis of somatic symptoms, depression, traumatic experiences and PTSD were not used in these subjects. PTSD was excluded in these individuals by using the Post-Traumatic Stress Symptom 10 Questionnaire (PTSS-10) [27]. Blood sampling in controls (10 ml) was performed between 8∶00 and 10∶00 h.

### Endocannabinoid Measurements

We used high-performance liquid chromatography in combination with tandem mass spectrometry (HPLC-MS-MS) to profile the plasma of trauma-exposed subjects and healthy controls for the following ECs and NAEs: ANA, 2-AG, PEA, OEA, SEA, OLDA, docosatetraenoylethanolamide (DEA), dihomo-γ-linolenoyl-ethanolamide (DLE) and N-arachidonoyldopamine (NADA). These compounds were selected because of their presence in human plasma [8], [28], [29], their responsiveness to acute stress [7], [10], [30], [31] or their cannabinomimetic activity [32]. Endocannabinoid measurements, including sample preparation and data interpretation, were performed at the Department of Anaesthesiology of the University of Munich in cooperation with the Institute of Doping Analysis und Sports Biochemistry, Dresden, Germany.

#### Extraction of the samples

Venous blood samples for EC measurements were taken and drawn into EDTA-containing tubes (BD Vacutainer, Franklin Lakes, NY, USA), centrifuged at 3,500 rpm, frozen at –30°C and stored at –80°C until assay. The time interval between blood sampling and centrifugation was kept below 10 min because previous experiments have shown that EC generation in blood samples is continued *ex vivo* [33], [34]. EC concentrations were determined within a few months after sampling. EC plasma levels in frozen samples have been shown to remain stable for at least 6 months [35]. For plasma extraction, we transferred 500 µl of plasma into a 2 ml reaction tube. Twenty microliter of internal standard was added consisting of the stable isotope-labeled ECs and NAEs ANA-d4, 2-AG-d5, PEA-d4, NADA-d8, SEA-d8, DEA-d4, DLE-d8 and OLDA-d8 (Cayman Europe, Tallinn, Estonia). The purity of these materials was >97.2%. Then 1 ml of methyl tertiary-butyl ether (MTBE, Sigma-Aldrich, Italy) was added and the mixture was vortexed for 30 s and centrifuged at 12,000×g for 6 min. The clear supernatant was transferred into a clean 5 ml polypropylene tube (Sarstedt, Numbrecht, Germany) and evaporated under N_2_ at 37°C. Dried organic phases were then reconstituted in 100 µl of acetonitrile, vortexed for 30 s, centrifuged at 12,000×g for 6 min and the clear solution was transferred to HPLC/MS-MS suitable tubes made of polypropylene.

#### High performance liquid chromatography-tandem mass spectrometry

All HPLC-MS-MS analyses were carried out using an 1100 LC system (binary pump and auto sampler, Agilent, CA, USA), coupled to an API 4000 mass spectrometer (AB Sciex, Darmstadt, Germany) equipped with a Turbo-Ion-Spray (ESI) source. The instrument software Analyst (version 1.5) was used for data processing. An Agilent Zorbax XDB-C18 analytical column (3 mm×150 mm, 5 µm particle size) was utilized for chromatographic separation. The mobile phase consisted of (A) 0.1% (v/v) formic acid (AppliChem, Germany) with 5 mM ammonium formate (diluted from a 10 M aqueous stock solution, Fluka, Germany) and (B) methanol (gradient grade) containing 5 mM ammonium formate buffer and 0.01% (v/v) formic acid. A linear gradient was employed at a flow rate of 700 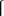
l/min from 55% B (at t = 0–1.2 min) to 100% B (t = 6–9 min), compatible with a source temperature of 550°C and source gas flow settings (nitrogen as sprayer and heater gas) of 50 psi. The injection volumes were 50 µl. Selected reaction monitoring (SRM) m/z transitions were: ANA = 348.2/311.3; 2-AG = 379.2/269.0; SEA 328.3/311.3; OEA = 326.3/95.1; PEA = 300.3/95.1 and OLDA = 418.3/154.2. For calibration, pure solutions of ANA (0.1–2 ng/ml), 2-AG (1.0–200 ng/ml), OEA (1.0–20 ng/ml), SEA (5–100 ng/ml), PEA (5–100 ng/ml), OLDA (5–100 ng/ml), DEA (1–5 ng/ml) and NADA (1–5 ng/ml) (Cayman Europe, Tallinn, Estonia) were applied. Our method is linear within these calibration ranges. The lower limit of detection of the method (defined as a signal/noise ratio >4∶1) was 0.025 ng/ml for ANA and 0.33 ng/ml for 2-AG. In biological matrices, 2-AG (including its deuterated analog) is rapidly isomerized to 1-AG [29], [36]. We therefore quantified 2-AG as the sum of 2-AG and 1-AG and refer to the sum of both compounds as 2-AG throughout the paper although this represents a chemical misnomer.

### Cortisol Measurements

Plasma cortisol levels were determined from the same samples that were used for EC measurements using an automated immunoassay system based on electrochemiluminescence as the principle of signal generation (Roche Elecsys, Roche Diagnostics, Mannheim, Germany). These analyses were performed at the Institute of Clinical Chemistry of the University of Munich following the manufacturer’s protocol.

The investigators who performed the measurements were blinded for the group assignment of the probes.

### Statistics

Data are presented as mean±SD, except in the figure where means±SEM is used to increase clarity. Deviation from normal distribution of sample data was tested using the Kolmogorov-Smirnov method. Plasma EC concentrations of healthy controls, trauma-exposed individuals without PTSD and subjects with evidence of PTSD were compared using analyses of variance (ANOVA) in normally distributed data and Kruskal-Wallis ANOVA on Ranks in case of non-parametric data, followed by an all pairwise multiple comparison procedure (Holm-Sidak or Tukey Test, respectively) as post-hoc test to determine which subgroups were significantly different from each other.

The relationship between continuous variables (e.g. EC levels and CAPS scores) in normally distributed data was quantified by calculating Pearson’s *r* and in case of a non-parametric distribution with Spearman’s *ρ*.

#### Power analysis for the required sample size based on effect size estimation

The main hypothesis to be tested in this study was that plasma EC and NAE concentrations in individuals with severe traumatic experiences are higher than in nontraumatized controls. We based our sample size estimation on a previous pilot study in cardiac surgical patients (n = 90) [19] because no other data on plasma EC concentrations in individuals with previous traumatic experiences and PTSD symptoms were available. In this study, patients with multiple traumatic experiences (e.g. from previous myocardial infarction or acute heart failure) had ANA plasma concentrations of 0.40±0.14 ng/ml [19]. Because baseline ANA plasma levels in healthy individuals measured with our method is in the range of 0.20 ng/ml, we assumed a detectable difference of 0.20±0.14 ng/ml between traumatized individuals and healthy controls. Based on an alpha value of 0.05 and a power of 0.80 and the comparison of 3 groups (healthy controls, subjects with traumatic experiences but no PTSD, and patients with PTSD) using ANOVA, we estimated a sample size of 8 per group to detect significant differences between groups. To be on the save side (with respect to possible dropouts, technical problems etc.) we aimed at 20 trauma-exposed individuals and at least 20 healthy controls.

## Results

### Demographics and Clinical Data

Trauma-exposed individuals and controls did not differ significantly with respect to age or gender distribution, but controls had more years of education. Individuals with PTSD reported a higher use of antidepressants than non-trauma-exposed controls. No significant differences were detected with regard to other types of medication (Table 1). In traumatized individuals, the number of cigarettes smoked/day correlated with CAPS sum scores (r = 0.505, p = 0.006). As expected, CAPS, HAMD and SOMS scores correlated positively with the number of traumatic event types experienced (CAPS: *r* = 0.70, p<0.01; HAMD: *r* = 0.71, p<0.01; SOMS: *r* = 0.55, p<0.01).

### Endocannabinoid Plasma Concentrations

**Figure 1:**
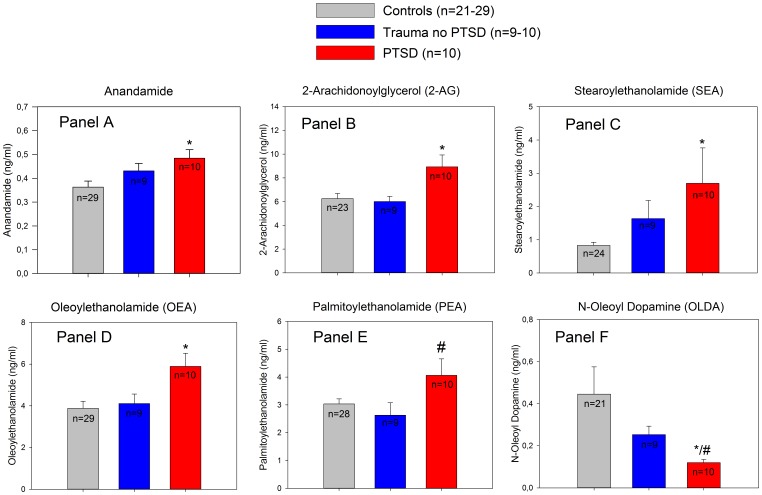
Plasma level comparisons of ECs and related NAEs between non-traumatized control subjects, trauma-exposed subjects and patients with PTSD. *Panel A*: ANA plasma concentrations; *significantly higher ANA concentrations in PTSD patients as compared to healthy controls (diff. of means = 0.120, t = 2.64, *p = 0.012; ANOVA with Holm-Sidak post hoc test). *Panel B*: 2-AG plasma levels; *significant difference compared to controls (diff. of means = 2.68, t = 3.12, unadjusted p = 0.003, critical level p = 0.017). *Panel C*: SEA levels: *significant difference compared to controls (Diff. of means = 1.87, t = 2.77, unadjusted p = 0.008, critical level p = 0.017). *Panel D*: *significantly higher OEA plasma levels in individuals with PTSD (diff. of means = 2.01, t = 3.03, unadjusted p = 0.004, critical level p = 0.017) compared to controls. *Panel E*: significant differences in PEA plasma concentrations compared to individuals after trauma exposure who did not develop PTSD (^#^p<0.05, diff. of ranks = 16.6, Q = 2.64, Kruskal-Wallis ANOVA on Ranks with Dunn’s post-hoc test). *Panel F*: significantly lower OLDA plasma concentrations compared to the control group (*p<0.05, diff. of ranks = 13.8, Q = 2.45) and in comparison to individuals after trauma exposure without PTSD (^#^p = 0.05, diff. of ranks = 12.40, Q = 2.75, Kruskal-Wallis ANOVA on Ranks with Dunn’s post-hoc test). Data are mean ± SEM.

EC and NAE plasma profiles in trauma-exposed individuals showed distinct differences between groups. Traumatized subjects with PTSD showed significantly higher plasma concentrations of ANA (0.48±0.11 vs. 0.36±0.14 ng/ml, p = 0.01), 2-AG (8.93±3.20 vs. 6.26±2.10 ng/ml, p<0.01), OEA (5.90±2.10 vs. 3.88±1.85 ng/ml, p<0.01), SEA (2.70±3.37 vs. 0.83±0.47, ng/ml, p<0.05) and significantly lower plasma levels of OLDA (0.12±0.05 vs. 0.45±0.59 ng/ml, p<0.05) than healthy controls. Moreover, PTSD patients had higher 2-AG (8.93±3.20 vs. 6.01±1.32 ng/ml, p = 0.03) and PEA plasma levels (4.06±1.87 vs. 2.63±1.34 ng/ml, p<0.05) than trauma-exposed individuals without evidence of PTSD (Figure 1).

Isotope-labeled DEA, DLE and NADA could be readily detected in the samples but no signals of native DEA, DLE or NADA could be found in trauma-exposed subjects or controls.

Because depression may influence the EC and NAE systems [7], and traumatized participants with PTSD had a higher incidence of major depression than traumatized participants without PTSD, we compared plasma EC/NAE levels between traumatized individuals diagnosed with depression or using antidepressants with individuals without these potentially confounding variables in a secondary analysis. We found no significant effect of depression or antidepressants on plasma EC/NAE levels (Table 2).

**Table 2:** Effect of comorbid depression or the use of antidepressants on plasma EC/NAE concentrations.

EC/NAE	Depression			Antidepressives		
	No (n = 10)	Yes (n = 9)	p	No (n = 14)	Yes (n = 5)	p
ANA (ng/ml)	0.46±0.08	0.46±0.14	0.88	0.48±0.09	0.40±0.16	0.13
2-AG (ng/ml)	7.16±2.30	7.90±3.64	0.64	7.95±3.10	6.43±1.51	0.32
PEA (ng/ml)	3.40±2.16	3.40±1.41	0.99	3.60±1.98	2.80±0.73	0.34
OEA (ng/ml)	4.75±1.77	5.30±2.28	0.58	5.25±2.11	4.67±1.24	0.45
SEA (ng/ml)	2.69±3.60	1.54±0.94	0.44	2.54±3.01	1.24±0.48	0.37
OLDA (ng/ml)	0.21±0.13	0.15±0.09	0.29	0.19±0.12	0.17±0.10	0.84

Trauma-exposed individuals who reported somatic comorbidities (n = 4) or the use of any type of medication except antidepressives (n = 5), had nearly identical plasma levels of ECs or NAEs compared to participants without any comorbidities or medication use (Table 3). Study participants including healthy controls who smoked (n = 11) tended to have higher EC/NAE concentrations than non-smokers (n = 37) but these differences were not statistically significant (Table 4). We found no statistically significant correlation between plasma concentration of EC/NAE compounds and the number of cigarettes smoked/day, neither in the whole study group (r≤0.155, p≥0.29, n = 48) nor when only trauma-exposed individuals with and without PTSD were included in the analyses (r≤0.312, p≥0.080, n = 19).

**Table 3:** Comorbidities (except depression) or medication use (except antidepressants) and plasma EC/NAE concentrations in individuals after trauma exposure.

EC/NAE	Comorbidities^a^			Medication use^b^		
	No (n = 15)	Yes (n = 4)	p	No (n = 14)	Yes (n = 5)	p
ANA (ng/ml)	0.47±0.10	0.42±0.15	0.41	0.46±0.11	0.49±0.10	0.35
2-AG (ng/ml)	7.44±2.60	7.92±4.12	0.77	7.42±2.90	7.89±3.00	0.76
PEA (ng/ml)	3.63±1.90	3.20±1.41	0.29	3.68±1.94	2.57±0.79	0.24
OEA (ng/ml)	4.90±1.63	5.20±3.03	0.48	5.15±1.94	4.75±2.10	0.71
SEA (ng/ml)	2.48±2.95	1.90±0.84	0.37	2.59±3.03	1.91±0.30	0.29
OLDA (ng/ml)	0.18±0.12	0.21±0.10	0.63	0.18±0.10	0.18±0.11	1.00

a Comorbidities were rheumatic disease (n = 1), hepatitis B (n = 1) and chronic gastritis (n = 2).

b Medications were contraceptives (n = 2), mood stabilizers (n = 1) and antacids (n = 2).

**Table 4:** EC/NAE concentrations in smokers vs. non-smokers.

EC/NAE (ng/ml)	Cigarette smoking		
	No (n = 37)	Yes (n = 11)	p
ANA (ng/ml)	0.38±0.13	0.47±0.10	0.12
2-AG (ng/ml)	6.70±2.20	7.22±3.35	0.57
PEA (ng/ml)	2.99±1.27	3.77±1.45	0.09
OEA (ng/ml)	4.12±2.04	4.94±1.51	0.25
SEA (ng/ml)	1.31±1.98	0.41±0.59	0.45
OLDA (ng/ml)	0.29±0.41	0.41±0.59	0.51

**Figure 2:**
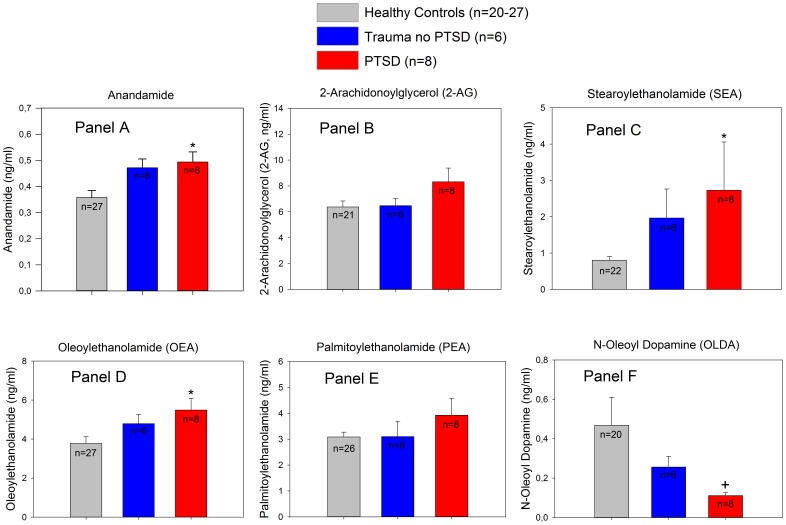
Subanalysis of plasma concentrations of ECs and related NAEs after excluding individuals of African descent (n = 7). *Panel A*: significantly higher ANA plasma concentrations in PTSD patients as compared to healthy controls (diff. of means = 0.136, t = 2.64, *p = 0.035; ANOVA with Holm-Sidak post hoc test). *Panel C*: significantly higher SEA plasma levels compared to controls (diff. of ranks = 12.9, Q = 2.96, p<0.05; Kruskal-Wallis ANOVA on Ranks with Dunn's post hoc test). *Panel D*: significantly increased OEA concentrations in PTSD patients in relation to healthy controls (diff. of ranks = 11.9, Q = 2.47, p<0.05; Kruskal-Wallis ANOVA on Ranks with Dunn's post hoc test). *Panel F*: OLDA concentrations across the 3 study subgroups (Kruskal-Wallis ANOVA on Ranks indicated a significant difference among the groups (^+^p = 0.02) but significance was lost after correction for multiple comparisons (diff. of ranks 11.7, Q = 2.23, p>0.05). The differences between groups shown in *Panel B* (2-AG) and *Panel E* (PEA) were not statistically significant in the subanalysis. Data are mean ± SEM.

Because it has recently been reported that the EC and NAE response to stress is different between Caucasians and individuals with an African genetic background [30], we performed a secondary analyses excluding non-Caucasian individuals (n = 7) (one each from Sudan, Namibia, Senegal and Nigeria and two from Gambia; two of these individuals were from the control group, 3 were trauma exposed but did not develop PTSD and 2 had PTSD). When non-Caucasians were excluded, differences in ANA, SEA and OEA in individuals with PTSD in relation to controls remained significant. OLDA levels remained also significantly lower but significance was lost after correction for multiple comparisons. 2-AG and PEA levels were still elevated in PTSD patients but the differences to healthy controls were no longer significant (Figure 2).

Because our control group was ethnically inhomogeneous and included refugees who did not report exposure to highly traumatic events nor had evidence of PTSD as well as individuals of German origin who may have a different health status or differences in the overall stress exposure, we performed additional analyses and excluded German subjects from the control group. In this subanalysis, individuals with PTSD showed still significantly higher 2-AG concentrations as compared to the ethnically more comparable controls (n = 9) (8.93±3.16 vs. 5.71±2.90 ng/ml, p = 0.02, ANOVA with Holm-Sidak post-hoc test) as well as significantly lower OLDA concentrations (0.12±0.05 vs. 0.19±0.08 ng/ml, p = 0.007).

**Figure 3:**
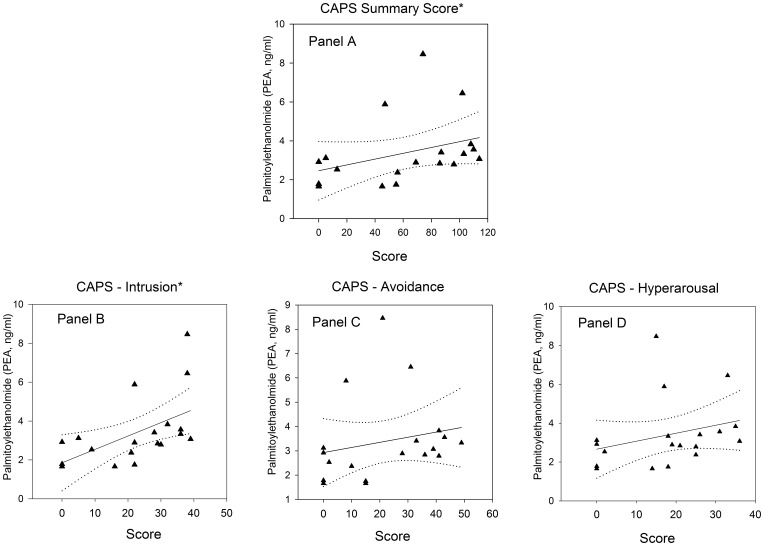
Correlation between CAPS scores and PEA plasma levels in individuals after trauma exposure (n = 19). Panel A: CAPS sum score (r = 0.54, p = 0.02); Panel B: CAPS – intrusion subscore (r = 0.65, p<0.01); Panel C: CAPS – avoidance subscore (r = 0.21, p = 0.40); Panel D: CAPS - hyperarousal subscore (r = 0.29, p = 0.23). Solid line indicates regression line and dotted lines 95% confidence intervals.*marks significant correlations.

**Figure 4:**
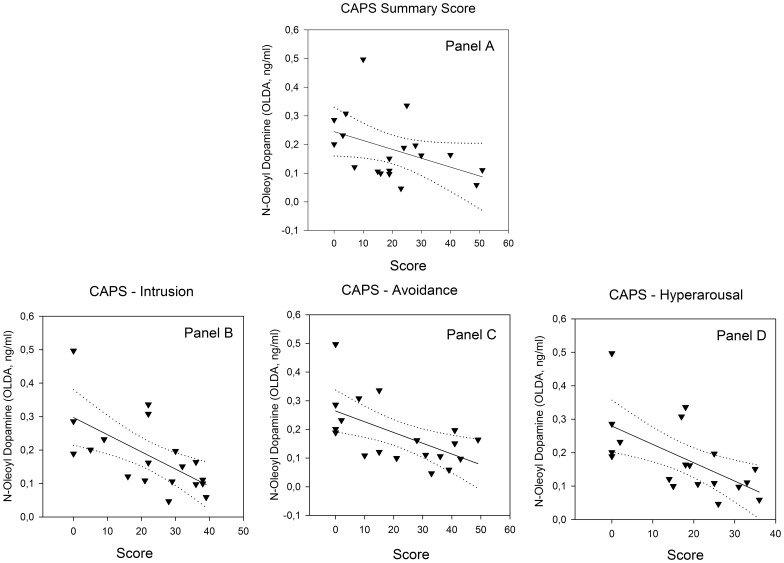
Relationship between OLDA plasma concentrations and CAPS scores. Panel A: CAPS sum score (r = −0.68, p<0.01, n = 19); Panel B: CAPS – intrusion subscore (r = −0.65, p<0.01); Panel C: CAPS – avoidance subscore (r = −0.59, p<0.01); Panel D: CAPS – hyperarousal subscore (r = −0.66, p<0.01). Solid line indicates regression line and dotted lines 95% confidence intervals.

**Figure 5:**
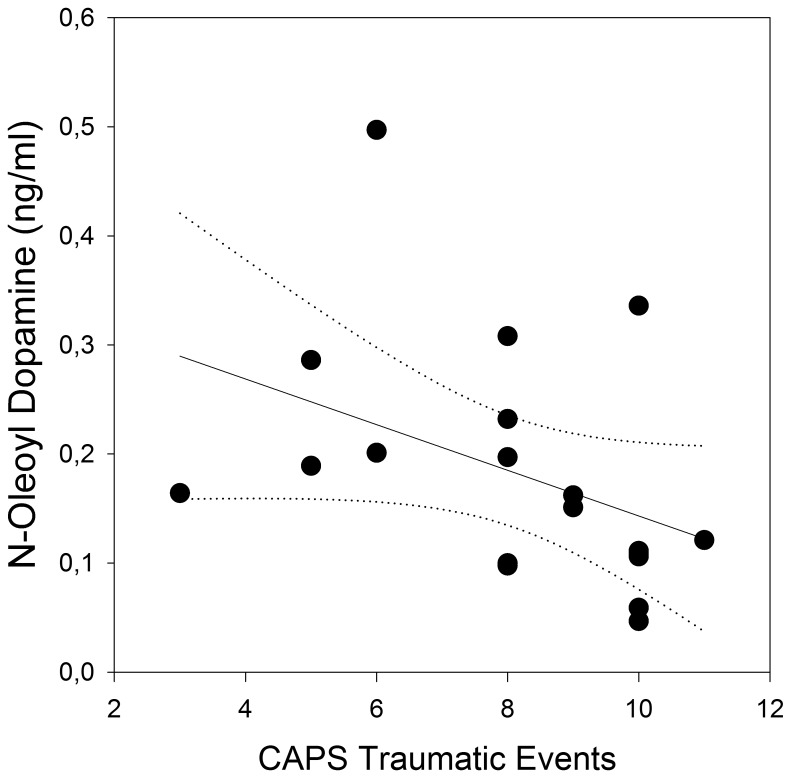
Correlation between the number of CAPS traumatic event types and OLDA plasma concentrations (r = −0.50, p = 0.03, n = 19). The solid line represents the regression line and dotted lines 95% confidence intervals.

Within the trauma-exposed group (n = 19) the CAPS sum score and the intrusion subscore correlated positively with PEA plasma levels (Figure 3). OLDA plasma levels were negatively related to the CAPS sum score, and all CAPS subscores correlated negatively with OLDA concentrations (Figure 4). Furthermore, there was a negative correlation between the number of reported CAPS traumatic events types and OLDA plasma levels (Figure 5) with no significant correlations between the other ECs/NAEs measured and traumatic event types (data not shown).

### Cortisol Plasma Concentrations

Cortisol concentrations in trauma-exposed individuals without PTSD were 14.0±7.0 µg/dl, 10.2±7.02 µg/dl in PTSD patients and 11.2±6.53 µg/dl in controls, but the difference between groups was not statistically significant (p = 0.45). There was also no significant correlation between the number of CAPS traumatic event types experienced, CAPS, SOMS, and HAMD scores, and plasma cortisol values. With the exception of a negative relationship between SAE plasma concentrations and cortisol levels in traumatized individuals (r = −0.55, p = 0.02, n = 19), we found no correlation between cortisol values and EC/NAE levels.

## Discussion

Our findings indicate that individuals with PTSD show significant differences in plasma concentrations of ECs and NAEs when compared to healthy controls and to subjects after trauma exposure who did not develop PTSD. Furthermore, we found significant correlations between CAPS scores and plasma concentrations of the NAEs PEA and OLDA in stressed, trauma-exposed individuals.

The responsiveness of the EC system in humans to *acute* stress has been demonstrated in several recent studies. Stress induced by the Trier Social Stress Test resulted in a significant increase in ANA, PEA and OEA plasma concentrations [30]. Physical stress in trained and physically fit individuals induced by strenuous exercise during mountaineering or cycling also resulted in elevated EC concentrations which returned to baseline after termination of the stressful activity [37], [38]. Exposure of healthy volunteers to kinetic stress during a parabolic flight experiment gave rise to a significant increase in plasma EC concentrations in stress-tolerant participants, whereas highly stressed individuals showed an absent EC response and a massive activation of the hypothalamic-pituitary-adrenal (HPA) axis [10]. Likewise, patients undergoing cardiac surgery who showed an impaired EC response during the stressful postoperative period were at an increased risk for depression at 6 months after surgery [39]. The latter two findings may allow speculating that an increased EC signaling during acute stress is a protective response to maintain homeostasis and enhance the recovery process. In line with this assumption, animal experiments showed that the systemic and intracranial injection of cannabinoids, resulting in an increase in EC signaling, prevented the effects of intense stress in animal models of PTSD [40]. The hypothesis that an activation of the EC system increases stress tolerance in humans needs to be verified in further studies.

Our chronically stressed individuals with PTSD showed sustained alterations in EC and NAE plasma concentrations which changed in proportion to PTSD symptom intensity and were seen long after termination of the actual life-threatening situation. Comparable observations have been made in the abovementioned pilot study in patients with severe heart disease awaiting cardiac surgery where patients with traumatic experiences and a high risk for PTSD showed significantly increased ANA and 2-AG plasma concentrations [19]. This persistently activated EC system could suggest the presence of a chronically increased allostatic load as a result of massive traumatic experiences. Although a short-term activation of the EC system during acute stress probably represents an adaptive and protective response of the organism [11], increased levels of ECs and NAEs as a result of chronic allostatic load might have both protective *and* harmful effects. The possible protective effects of an activated EC system are illustrated by the fact that patients with major depression show reduced serum ANA and 2-AG concentrations [7] and that the use of the CB1 receptor antagonist rimonabant as an anti-obesity drug has been associated with a significant increase in the risk for depression. This effect was particularly pronounced when individuals taking rimonabant faced major stressful events [5]. Interestingly, patients with PTSD often abuse cannabinoids [16] and the use of the synthetic cannabinoid nabilone resulted in an improvement of symptoms [17]. Thus, despite elevated EC and NAE plasma concentrations as part of a stress reaction in PTSD patients, the enhanced endocannabinoid signaling might be insufficient to completely restore homeostasis and a further reduction in symptoms by exogenous cannabinoids is possible. A likely explanation for this phenomenon is that the chronic elevation of EC and NAE levels in traumatized individuals could result in a desensibilisation or downregulation of one or more receptor types for ECs or NAEs. This possibility has been described in individuals with intolerance to acute stress and an inadequate EC response during a parabolic flight experiment [10]. Although an enhanced EC signaling may have protective and adaptive effects during or after a major stress exposure, there is evidence of elevated plasma levels of ANA and 2-AG in patients with atherosclerosis and coronary artery disease [20], [41], whereas an inhibition of EC signaling by CB1 receptor blockade in circulating macrophages is associated with a significant reduction in pro-inflammatory mediators [20]. Activated macrophages and other nuclear blood cells are believed to be the major source of circulating ECs in blood [33], [42]. Therefore, an activated peripheral EC system might play a causal role in the reported increased risk for coronary artery disease and the associated enhanced mortality in patients with PTSD [43]. This assumption is corroborated by the observation that the number of cigarettes/day smoked by traumatized individuals in our study correlated with CAPS summary scores and that smokers tended to exhibit higher EC/NAE plasma concentrations, albeit without a direct correlation between EC/NAE concentration and the amount of daily tobacco consumption. One could therefore argue that the increased activity of the EC system in traumatized individuals observed in our study could help to maintain homeostasis in the short term but might have long-term adverse consequences. These assumptions need, however, experimental and clinical confirmation.

The interpretation of our findings is seriously hampered by the fact that little detailed knowledge exists regarding the multiple biological effects of many of the only very recently discovered ECs and NAEs measured in our study. Furthermore, the mechanisms leading to changes in peripheral EC and NAE concentrations are at present only incompletely understood and the important question whether changes in EC and NAE signaling reflect pathological or protective responses of the organism after trauma exposure is difficult to address.

Other limitations of our study result from the fact that we measured peripheral plasma concentrations of ECs/NAEs whereas most effects of lipid signaling molecules on stress and memory are central [13]–[15], [44]. There is, however, evidence that ECs and related compounds are able to cross the blood-brain barrier [45]. Furthermore, recent animal experiments have demonstrated the possibility of a parallel activation of the peripheral and central EC system during stressful conditions. The stress of footshock delivery to rats resulted in an almost simultaneous increase in ANA concentrations in blood and several memory–related brain areas including the amygdala and hippocampus (Hauer D., Campolongo P. et al., in preparation). The systemic administration of OEA to rats, resulting in elevated OEA plasma levels as seen in our PTSD patients, improved memory of aversive training experiences [46]. These findings suggest that changes in peripheral EC/NAE concentrations may indeed reflect alterations in central EC signaling along with EC/NAE associated behavioral effects. One should keep in mind, however, that animal studies have also shown that stress *reduces* brain EC concentrations [44]. Thus, the source and regulation of circulating ECs/NAEs during stress needs to be addressed in further studies.

Additional limitations of our study result from ethnical differences within the subject pool we investigated. The individuals we studied had different racial backgrounds and included Caucasians and Africans. A recent study has shown that the lipid response to acute stress is significantly higher in Caucasians than in African-Americans [30]. Although these findings cannot necessary be extrapolated to chronic trauma-induced stress conditions, we performed an additional exploratory subanalysis excluding individuals of African origin. Despite the loss of statistical power by this approach, higher plasma EC concentrations in PTSD patients could be confirmed in this more homogenous sample and the group differences in ANA, SEA and OEA remained significant whereas OLDA concentrations still showed a strong trend towards lower values. In order to control for our inhomogeneous control group which included individuals of German origin as well as immigrants with refugee status in Germany, we further compared EC/NAE plasma concentrations among subgroups with German subjects excluded from controls. In this more uniform sample, individuals with PTSD still showed significantly higher plasma concentrations of 2-AG and significantly lower levels of OLDA. Beside the lower statistical power due to the smaller control group, the loss of significance in ANA, SEA and OEA was due to increased EC/NAE levels in the remaining controls. These individuals did not report exposure to highly traumatic events nor had evidence of PTSD but were still refugees who found shelter in Germany. They were almost certainly exposed to higher stress levels during immigration which could result in increased plasma lipid signaling, although this possibility is difficult to proof with the questionnaires and instruments used in our study. Thus, when compared to our original control group, individuals with PTSD showed significantly higher plasma concentrations of ANA, 2-AG, SEA and OEA and significantly lower levels of OLDA. When individuals of African origin were excluded, ANA, SEA and OEA remained significantly elevated; 2-AG and PEA levels were still higher and OLDA concentrations still lower but statistical significance was lost with regard to the latter three compounds. When only refugees with presumed differences in stress exposure were compared, only 2-AG levels remained significantly higher and OLDA concentrations significantly lower. This suggests that these two compounds may represent more robust indicators of the presence of PTSD but these assumptions definitively need to be corroborated in larger studies.

It is of interest to note that plasma concentrations of ECs and NAEs in trauma-exposed individuals who did not develop PTSD were often between those of controls and those of subjects with the full syndrome of PTSD. This suggests stress-dose dependent changes in lipid signaling which is also corroborated by our observation that CAPS and CAPS subscores correlated with EC/NAE plasma levels.

Individuals who developed PTSD had experienced a significantly higher number of war and torture events than traumatized subjects without PTSD and this raises the possibility that the greater intensity of the original stress exposure, rather than PTSD itself, may be the actual cause of the observed differences in our study. However, we found no significant relationship between the number of traumatic event types reported by our study subjects and EC/NAE plasma concentrations, which correlated only with PTSD symptom severity.

Study participants with PTSD had a higher incidence of depression and also reported a higher use of antidepressants. The association between PTSD and depression is, however, complex. Depression is known to be associated with *lower* EC plasma levels [7], [39] whereas our individuals with PTSD showed *increased* concentrations. Moreover, our secondary analyses showed no differences in EC/NAE plasma concentrations in traumatized individuals on antidepressants and between depressed and non-depressed individuals. Thus, if depression and the use of antidepressants have an effect on our finding of increased EC/NAE plasma levels in PTSD patients one could assume that these confounders may have actually resulted in lower rather than increased plasma concentrations. Nevertheless, the role of depression in EC signaling in the presence of PTSD definitively needs further studying in larger cohorts.

PTSD patients may show higher stress responsiveness than healthy controls and could thus react more to the stress of blood sampling, which could result in higher EC/NAE plasma levels. These effects are difficult to control for, but all our subjects appeared to be comfortable during blood drawing and were habituated to the procedure from previous medical visits.

Despite these limitations, our findings indicate that PTSD is associated with changes in the pattern of plasma EC/NAE concentrations. This finding may have both pathophysiological as well as diagnostic consequences for PTSD but needs to be reproduced in larger and more homogenous study groups. Likewise, early (e.g. during childhood) trauma exposure may have very different effects on the EC system than later in life and this possibility should also addressed in further studies.
